# Exercise Habits and BMI in Pediatric Patients with Anomalous Aortic Origin of Coronary Arteries

**DOI:** 10.3390/children13070948

**Published:** 2026-07-20

**Authors:** Thomas S. Przybycien, Dana Reaves-O’Neal, Kimberly Gray, Sandra Mihail, Tam T. Doan, Shagun Sachdeva, Ziyad M. Binsalamah, Silvana Molossi

**Affiliations:** 1 Department of Pediatrics, Texas Children’s Hospital, Baylor College of Medicine, Houston, TX 77030, USA; 2 Coronary Artery Anomalies Program, Texas Children’s Hospital, Houston, TX 77030, USA; 3 The Lillie Frank Abercrombie Section of Cardiology, Texas Children’s Hospital, Baylor College of Medicine, Houston, TX 77030, USA; 4 School of Medicine, Baylor College of Medicine, Houston, TX 77030, USA; 5 Maine Health Cardiovascular Surgery, Portland, ME 04102, USA

**Keywords:** BMI, exercise, congenital heart disease, anomalous aortic origin of coronary artery

## Abstract

**Highlights:**

**What are the main findings?**

- One third of patients with AAOCA are obese or overweight.
- Those who underwent surgery are more likely to exercise competitively.

**What are the implications of this main finding?**

- Exercise choices in children with AAOCA represent a balance between clinical risk and a healthy lifestyle.
- Future studies are needed to determine optimal exercise recommendations in AAOCA.

**Abstract:**

**Background:** Exercise habits are unknown in pediatric patients with anomalous aortic origin of coronary arteries (AAOCA). In this study, we aim to identify differences in exercise habits and their relationship to BMI and other demographic factors in pediatric patients with AAOCA. **Methods:** A single-center, cross-sectional study of 573 patients with AAOCA (mean age = 10.5 years, SD 6.3, 63% males) was conducted. Exercise habits were classified as recreational or organized/competitive. BMI percentiles were stratified as normal (5th to ≤85th), overweight (85th to ≤95th), and obese (≥95th). Exercise habits and BMI were compared by demographic factors, presence of ischemia, and surgery status. Means were compared using two-tailed *t*-tests and multivariate linear regression with a false discovery rate correction. χ^2^ tests were used for categorical variables. **Results:** Nearly two-thirds (63.5%) of patients exercise recreationally, and 35% of the total cohort are overweight or obese (BMI > 85th%ile). Those who exercise competitively (29%) were more likely to have had surgery (24% vs. 15%, *p* = 0.019) and less likely to be exercise-restricted (3% vs. 8.5% *p* = 0.018). BMI in right-AAOCA is significantly elevated compared to left-AAOCA (63.1 vs. 53.1 percentile, *p* = 0.018). There was no difference in BMI percentile in those restricted from exercise or in those who exercised competitively. BMI and exercise habits are unrelated to ischemia, AAOCA subtype, exercise habits or restriction on multivariate analysis. **Conclusions:** One-third of AAOCA patients are overweight or obese, and BMI is not associated with exercise habits in this cohort. Surgical patients were more likely to exercise competitively, and this may have implications for personal choices to engage in exercise. Future studies are needed to determine optimal exercise recommendations to promote active lifestyles while minimizing the risk of sudden death.

## 1. Introduction

Anomalous aortic origin of coronary arteries (AAOCA) remains a leading cause of sudden cardiac death (SCD) in youth [1,2,3,4]. While most patients are asymptomatic prior to diagnosis, on rare occasions some patients’ first symptoms are sudden cardiac arrest/death (SCA/SCD) or arrhythmia [5]. Risk stratification of SCA/SCD remains a challenge in the young AAOCA population. In patients with diagnosed AAOCA, exercise recommendations are largely guided by their risk of ischemic injury/arrhythmia given their underlying anatomy (higher risk left (L)-AAOCA versus lower risk right (R)-AAOCA) and inducible ischemia on provocative stress testing. Given that exercise can precipitate SCA/SCD, it is common practice to restrict exercise in patients with high-risk anatomy or documented ischemia on advanced cardiac imaging [6,7,8,9,10]. Currently, there is no universal standardized practice guideline for exercise restriction in those with AAOCA, and questions regarding the long-term risk-benefit balance of exercise restriction and cardiovascular health and BMI are not yet understood. Moreover, it is not yet established how current exercise habits and exercise restriction in those with AAOCA impact patient BMI. Although roughly 30% of children with congenital heart disease are overweight or obese, only two small studies have examined exercise restriction and BMI in the AAOCA population [11,12,13,14]. However, of those who were not exercise restricted, no previous study to our knowledge has examined the actual exercise habits of AAOCA patients and their relationship to BMI, evidence of ischemia, and demographic factors. Given the established association between BMI and lack of exercise, we aim to explore differences in exercise habits, restriction, and BMI in pediatric patients with AAOCA in a hypothesis-generating study.

## 2. Methods

### 2.1. Study Population

This is a single-center, one-point-in-time, cross-sectional study of 573 patients, all ≤20 years old, with AAOCA. All patients were previously prospectively enrolled in our longitudinal registry at the Texas Children’s Hospital’s Coronary Artery Anomalies Program between December 2012 and April 2023. The Institutional Review Board approved our protocol (IRB H-32955 and H-43968, last approved in May 2026). Patients with complex congenital heart disease or hypoplastic coronaries with normal origin were not included in this cohort.

### 2.2. Clinical Evaluation

Evaluation, clinical decision-making, and follow-up of patients with AAOCA followed our institutionally standardized algorithm with the best available evidence that has been previously published [5,15,16]. Patient demographics and the mode of clinical presentation (ER vs. outpatient referral, electrocardiogram, echocardiogram, computed tomography angiography, exercise stress test and advanced imaging with provocative stress) were collected for all patients as previously described [17]. Exercise habits were classified as recreational or organized/competitive at the most recent follow-up via a qualitative standardized questionnaire in our electronic medical record. Specifically, those participating in organized in-school or out-of-school sports were classified as organized/competitive. All others were classified as recreational due to the ambiguity between truly sedentary patients and those who are more active at school recess or in physical education class. Quantitative measures of exercise (such as metabolic equivalents) were not used due to the ambiguity of exercise type, effort and duration in young children.

### 2.3. Statistical Analysis

BMI percentiles were stratified using the CDC boys’ and girls’ 2–20 growth curve as normal (5th to ≤85th%ile), overweight (85th to <95th%) and obese (≥95th%ile) as well as converted to a standardized CDC BMI z-score. BMI and exercise habits were collected at the most recent follow-up clinic visit. Exercise habits were compared across demographic parameters (self-reported race/ethnicity and gender) and clinical characteristics: the subtype of AAOCA (left (L) versus right (R)), the presence of ischemia, exercise restriction, and surgery status. Continuous variables were evaluated for normality using Kolmogorov–Smirnov tests. Normally distributed variables were expressed as mean (standard deviation) and categorical variables as number (percentage). Two-tailed *t*-tests were used for normally distributed variables (age at diagnosis and BMI) and the χ^2^ test was used for categorical variables. Multivariate linear regression modeling was performed to explore the associations of exercise habits with BMI when accounting for surgical status, the presence of myocardial ischemia, exercise restriction, the AAOCA subtype, and race/ethnicity. BMI z-scores were used to control for age and sex. Due to the cross-sectional nature of our data, our multivariate regression reflects demographic and disease associations with BMI, not predictors. A false discovery rate (FDR) correction was applied post hoc due to multiple comparisons. Statistical significance was defined as a 2-sided α < 0.05. Statistical analysis was performed using SPSS version 29 (IBM SPSS Inc., Chicago, IL, USA).

## 3. Results

### 3.1. Study Population

**Figure 1:**
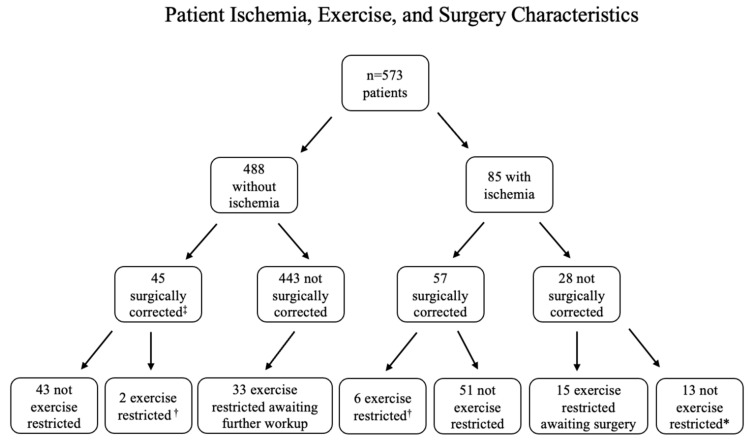
Patient distribution with respect to ischemia, surgery status and exercise restriction. At the time of data collection, 15% of the cohort had evidence of ischemia on advanced imaging and 17.8% underwent surgery. Restriction from exercise was counseled in 9.8% of the total cohort, all awaiting further testing to complete evaluation. * Family preference without symptoms during exercise, borderline stress testing. ^†^ Post-op recovery awaiting stress testing. ^‡^ Due to high-risk features and family preference.

The mean age at diagnosis was 10.5 (SD 6.3) years, with 62.7% of patients being male (Table 1). Race and ethnicity were similarly distributed to our population in the Greater Houston Area, with 38% being of Hispanic origin, 28.4% non-Hispanic Black, and 27.4% non-Hispanic White [18]. Most patients (77.8%) had a diagnosis of R-AAOCA, while 13.1% had L-AAOCA, 6.3% had a single coronary artery, and 2.8% had other AAOCA subtypes (Table 1). All 85 (14.8%) patients with demonstrated ischemia on advanced imaging were restricted from exercise at their initial clinic visit (Figure 1). One hundred two (17.8%) underwent surgical repair of AAOCA due to evidence of ischemia on testing, presentation with SCA or significant symptoms concerning for ischemia. Fifty-seven of 85 (67%) patients with demonstrated ischemia underwent surgical repair, and 45 of 488 (9%) of those without ischemia underwent surgical repair due to high-risk anatomic features and family preferences in shared decision-making. Of the remaining 28 patients with evidence of ischemia who had not undergone surgical repair, 15 were restricted from exercise awaiting surgery, and 13 were not restricted given shared decision-making with the family in the setting of a lack of exertional symptoms. At the time of data collection, 56 (9.8%) patients of the total cohort were restricted from exercise. Of these, 15 with documented ischemia were awaiting surgery, eight were awaiting completion of post-operative studies, and 33 were pending future pre-operative studies.

### 3.2. Exercise Habits and BMI

Most patients (63.5%, 363/573) exercised recreationally, and 29.1% (167/573) exercised in organized or competitive sports (Table 1). Forty-three (7.5%) patients did not have documented exercise habits. All 573 patients had BMI data available. The mean BMI percentile was 62 (SD 31.5). The mean BMI z-score was 0.46 (SD 1.2). Most patients (60.4%) had BMI percentiles in the normal range (5th to <85th%ile) while 15.7% had a BMI in the overweight range (85th to <95th%ile) and 19.4% had a BMI in the obese range (≥95th%ile).

When stratifying by exercise habits, those in organized or competitive sports were diagnosed at a later age (9.6 vs. 11.9 years old, *p* = <0.001, 95% CI 1.29–3.2) (Table 2). Moreover, those who exercised competitively were significantly more likely to have undergone surgical correction for AAOCA [χ^2^(df = 1, n = 529) = 5.54, *p* = 0.019] and less likely to be exercise restricted ([χ^2^(df = 1, n = 530) = 5.56, *p* = 0.018]. There was no difference in BMI across exercise habits (Table 2).

Furthermore, there was no difference in race/ethnicity, diagnosis (L-AAOCA vs. R-AAOCA) or the presence of ischemia between those who exercised recreationally vs. organized/competitively.

**Figure 2:**
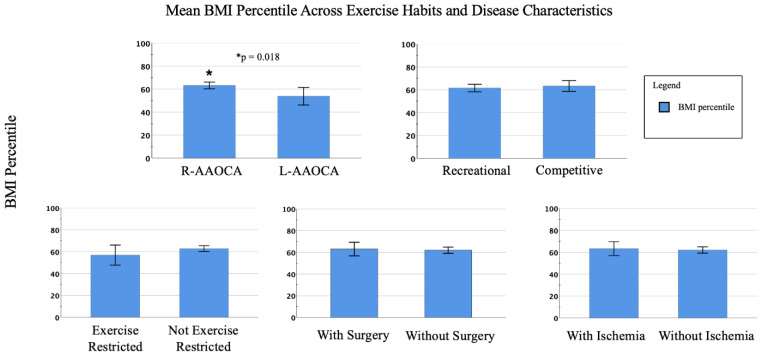
Body mass index percentile means compared across exercise habits, level of exercise, AAOCA type, exercise restriction, surgical repair, and evidence of ischemia. Error bars represent standard deviation of the mean. Abbreviations: BMI: body mass index, L-AAOCA: anomalous aortic origin of the left coronary artery, R-AAOCA: anomalous aortic origin of the right coronary artery.

Patients with R-AAOCA had a significantly higher BMI than those with L-AAOCA (63.1%ile vs. 53.1%ile, * *p* = 0.018) (Figure 2). However, there was no difference in BMI across exercise habits, surgery status, the presence of myocardial ischemia or exercise restriction status (Figure 2). When stratifying by the presence or absence of myocardial ischemia, there was no difference in BMI percentiles between those who exercised recreationally vs. organized/competitively (Supplemental Figure S1).

Regression analysis, accounting for differences in age, sex and surgical status at the time of BMI measurement, revealed no association between the level of exercise on BMI (Table 3). Moreover, while those with L-AAOCA seemed to have a lower BMI than those with R-AAOCA, this difference was not significant after false discovery rate correction (*p* = 0.186 95% CI −0.696, −0.033). Similarly, the evidence of ischemia, race/ethnicity, surgical status, and exercise restriction were not associated with significant changes in BMI.

## 4. Discussion

To our knowledge, this is the largest study to date to evaluate BMI and exercise habits in pediatric patients with AAOCA. Given the increasing prevalence of pediatric obesity, with nearly 20% of pediatric patients having a BMI in the obese range, our findings are consistent with national data in young patients with congenital heart disease [13,14,19]. It is unclear why patients with R-AAOCA had significantly higher BMI percentiles when compared with those with L-AAOCA, particularly when the L-AAOCA population is traditionally restricted from exercise more than the R-AAOCA, though this may relate to the higher prevalence of R-AAOCA compared to L-AAOCA (5 times higher in our study) [20]. There was no difference in age at diagnosis between R-AAOCA and L-AAOCA to explain the BMI differences (*p* = 0.617 95% CI −1.84, 1.10). Moreover, this association did not endure after false discovery rate correction in our regression model. Interestingly, the presence or absence of ischemia did not alter BMI percentiles in patients based on their exercise habits in univariate and multivariate regression analyses, suggesting that ischemia even after intervention, does not impact BMI. Older patients at diagnosis were more likely to exercise competitively, which is consistent with our clinical experience that many early teenagers are diagnosed either after referral for a spurious murmur or for chest pain during sports.

Furthermore, patients who underwent surgical repair were more likely to exercise competitively. In our clinical experience, this is an expected finding given the patient and familial desire to return to competitive play. Recently, our group published data indicating that 35% of children with AAOCA self-restricted from exercise despite not being restricted by their primary pediatric cardiologist [21]. Moreover, 61% of parents reported feeling that their child was unsafe to exercise competitively [21]. Notably, 47% of children desired to exercise more, and we hypothesize that surgical correction of their AAOCA may have a positive effect on their willingness to exercise competitively or return to competitive sports, although this can only be determined through future longitudinal studies.

Patients who exercised competitively were less likely to be restricted from exercise. At our institution, children are routinely restricted if they have documented ischemia, a previous cardiac arrest, arrhythmia or high-risk features [1]. Given our institutional practice of shared decision-making with families, this may account for athletes’ preferences to continue exercising competitively despite the known risks of exercise in AAOCA. This is in line with the most recent American Heart Association/American College of Cardiology Scientific Statement for competitive athletic participation in those with R-AAOCA, which allows asymptomatic patients with low-risk anatomy R-AAOCA a normal exercise stress testing, and an absence of ischemia on perfusion studies to be allowed to compete in athletics [20,22].

We identified only two other studies evaluating exercise restriction and BMI in patients with AAOCA, both of which were notably smaller than our cohort [11,12]. Exercise habits and exercise restriction in our population did not detrimentally impact BMI, which is consistent with the previous two longitudinal studies [11,12]. Specifically, Elias et al. 2017 demonstrated no difference in BMI between patients who were exercise restricted and those who were not, over a mean follow-up of 3.6 years [11,12]. Our data, along with Elias et al. 2017 [11], suggest that BMI is not related to exercise habits/restriction in the AAOCA population as was previously thought, but this requires further exploration with longitudinal data. Regarding exercise restriction, roughly 10% of our cohort was exercise restricted at the time of the last clinic follow-up. When compared with data from the Congenital Heart Surgeons’ Society (CHSS) AAOCA Registry, where Meza et al. 2017 reported that 55% of patients were exercise restricted [12], our cohort had a significantly lower restriction rate. Similarly, most patients (82%) were exercise restricted at baseline in the study by Elias et al. 2017 [11]. Given that our data was collected at the most recent follow-up, even after accounting for those who were awaiting further workup (33) and those with documented ischemia (85), only 20.5% would have been restricted at baseline, which is far less than reported in the comparable literature. This may partially be accounted for by the fact that these studies are older and recent guidelines are more permissive for those with R-AAOCA (without ischemia and low-risk anatomy) to exercise competitively [22]. This difference can likely be accounted for by family preferences in shared decision-making and our evolved understanding of the risks of AAOCA and exercise, as those smaller studies were conducted seven years ago.

Our retrospective, cross-sectional study does have limitations. Elevated childhood obesity rates in our study may be impacted by our USA patient population when compared with other countries or regions. Firstly, it is challenging to determine whether reported exercise habits truly reflected the patients’ actual level of activity. For instance, one may be a part of a competitive sports team but not be a starting player or miss numerous practices. Also, given the perception of exercise safety in this population as recently reported by Mihail et al. [21], even those without exercise restriction may not be participating at their full capacity. Moreover, the differences between recreational and competitive athletics are often unclear. There was collinearity risk in our regression model given that exercise habits and restriction were both included in the model. Importantly, variance inflation factor testing to detect collinearity was negative for our data. Moreover, the time from diagnosis to the last follow-up was 4.4 years (SD 3.1 years). While surgical status was controlled for in the regression model, some patients who were recently operated on may still be restricted, compared with those who had surgery years ago and may have returned to competitive sports. This may have introduced a selection bias. Lastly, at our Coronary Artery Anomalies Program, many patients present for second opinions or consultations from across the USA and then return to their home institutions, thereby making longitudinal follow-up of both BMI and exercise habits/restriction challenging.

Future studies evaluating differences in BMI longitudinally in pediatric patients with AAOCA are warranted, given that many are restricted from exercise, rising childhood obesity, and overall variable exercise habits. In addition, a deeper understanding of the risk associated with exercise in patients with AAOCA is needed to better determine exactly which patients are at risk for severe cardiovascular events and to ensure their safety during exercise.

## Figures and Tables

**Table 1:** Patient demographics.

Mean (SD) or Number (%) n = 573	
Age at diagnosis	10.5 (6.3)
Gender (M)	359 (62.7%)
Race/Ethnicity	
Hispanic	218 (38%)
Non-Hispanic White	157 (27.4%)
Non-Hispanic Black	163 (28.4%)
Asian	26 (4.5%)
Native American	2 (0.3%)
Unknown	7 (1.2%)
Diagnosis	
R-AAOCA	446 (77.8%)
L-AAOCA	75 (13.1%)
Single Coronary Artery	36 (6.3%)
Other *	16 (2.8%)
Evidence of Myocardial Ischemia (Y)	85 (14.8%)
Coronary Surgical Repair (Y)	102 (17.8%)
Exercise Habits	
Recreational	363 (63.5%)
Organized	111 (19.3%)
Competitive	56 (9.8%)
Unknown	43 (7.5%)
Exercise Restriction (Y)	56 (9.8%)
BMI z-score	0.46 (1.2)
BMI %ile	62 (31.5)
BMI < 5%ile	26 (4.5%)
BMI 5% to <85%ile	346 (60.4%)
BMI 85% to <95%ile	90 (15.7%)
BMI ≥ 95%ile	111 (19.4%)

* 12 anomalous circumflex, 1 RCA atresia, 2 LMCA atresia, 1 R-AAOCA not fully characterized by initial imaging.

**Table 2:** Exercise habits compared to patient demographics, BMI, and disease characteristics. Means were compared using two-tailed *t*-tests. χ^2^ tests were used for categorical variables. * *p* value ≤ 0.05.

Recreational Exercise Habits Mean (SD) or Number (%) n = 363		Organized or Competitive Exercise Habits Mean (SD) or Number (%) n = 167		*p* Value
Age at diagnosis	9.6 (6.7)	Age at diagnosis	11.9 (4.2)	<0.001 *
Gender (M)	229 (63%)	Gender (M)	101 (60%)	0.78
Race/Ethnicity		Race/Ethnicity		
Hispanic	141 (41.2%)	Hispanic	61 (39.1%)	0.73
Non-Hispanic White	96 (28%)	Non-Hispanic White	47 (30.1%)	0.75
Non-Hispanic Black	105 (30.8%)	Non-Hispanic Black	48 (30.8%)	0.97
Diagnosis		Diagnosis		
R-AAOCA	275 (85.1%)	R-AAOCA	134 (85.9%)	0.68
L-AAOCA	48 (14.9%)	L-AAOCA	22 (14.1%)	0.86
Evidence of Myocardial Ischemia (Y)	50 (13.7%)	Evidence of Myocardial Ischemia (Y)	27 (16.2%)	0.47
Coronary Surgical Repair (Y)	56 (15.4%)	Coronary Surgical Repair (Y)	40 (24%)	0.019 *
Exercise Restriction (Y)	31 (8.5%)	Exercise Restriction (Y)	5 (3%)	0.018 *
BMI %ile	61.4 (31.8)	BMI %ile	63 (30.6)	0.52
BMI < 5%ile	19 (5.2%)	BMI < 5%ile	6 (3.6%)	0.42
BMI 5% to <85%ile	215 (59.2%)	BMI 5% to <85%ile	104 (62.2%)	0.74
BMI 85% to <95%ile	63 (17.3%)	BMI 85% to <95%ile	22 (13.1%)	0.29
BMI ≥ 95%ile	66 (18.2%)	BMI ≥ 95%ile	35 (20.9%)	0.53

Abbreviations: BMI, body mass index, SD, standard deviation, R-AAOCA, anomalous aortic origin of the right coronary artery, L-AAOCA, anomalous aortic origin of the left coronary artery.

**Table 3:** Linear regression of patient demographics and disease characteristics on their association with BMI z-scores with unadjusted and corrected *p* values for false discovery rate. R^2^ = 0.033, n = 445. * *p* value ≤ 0.05.

Disease or Demographic Characteristic	β	95% CI	*p* Value (Unadjusted)	*p* Value (FDR)
Exercise Habits: Organized/Competitive vs. Recreational	0.081	−0.17, 0.33	0.528	0.528
Race/Ethnicity: Hispanic	0.283	−0.003, 0.57	0.053	0.186
Race/Ethnicity: Black	0.168	−0.16, 0.46	0.282	0.522
L-AAOCA vs. R-AAOCA	−0.364	−0.69, −0.03	0.032 *	0.186
Coronary Surgical Repair (Y)	0.134	−0.28, 0.44	0.447	0.522
Evidence of Myocardial Ischemia (Y)	−0.172	−0.57, 0.20	0.376	0.522
Exercise Restriction (Y)	−0.219	−0.78, 0.22	0.377	0.522

CI: Confidence Interval, FDR: false discovery rate.

## Data Availability

No datasets were generated or analyzed during the current study.

## Supplementary Materials

The following supporting information can be downloaded at: https://www.mdpi.com/article/10.3390/children13070948/s1, Figure S1: Body mass index percentile means compared across exercise habits (recreational versus organized/competitive) and stratified by the presence or absence of myocardial ischemia on advanced imaging. Error bars represent standard deviation of the mean. BMI: Body mass index.
