# Supplementary material for: Exercise Habits and BMI in Pediatric Patients with Anomalous Aortic Origin of Coronary Arteries
Source: Children (Basel). 2026 Jul 20;13(7):948. doi: 10.3390/children13070948 (PMC13406908; doi:10.3390/children13070948)
Supplement: Supplementary file 1 [file children-13-00948-s001.zip › children-4393056-supplementary.pdf]

Figure S1: Body mass index percentile means compared across exercise habits (recreational versus organized/competitive) and stratified by the presence or absence of myocardial ischemia on advanced imaging. Error bars represent standard deviation of the mean.

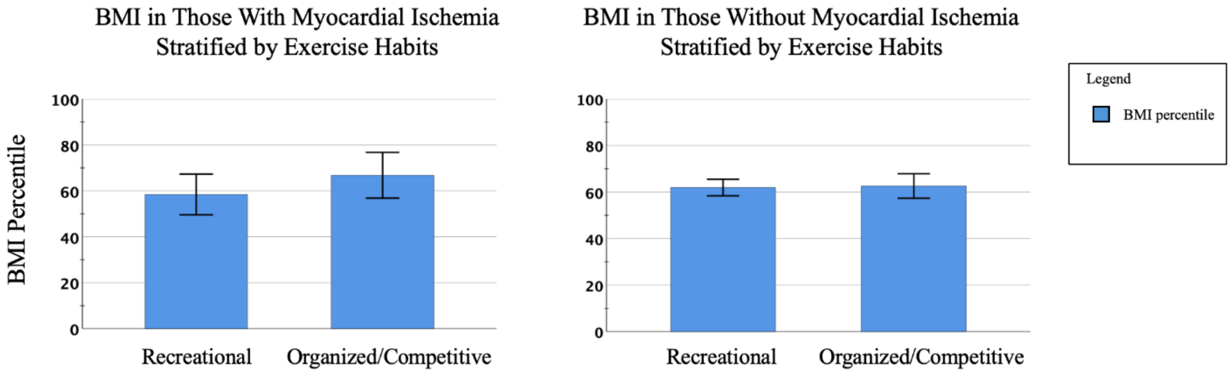

BMI: Body mass index.
